# Dysregulation of cardiac lipid parameters in high-fat high-cholesterol diet-induced rat model

**DOI:** 10.1186/s12944-018-0905-3

**Published:** 2018-11-14

**Authors:** Qian Han, Sze C. Yeung, Mary S. M. Ip, Judith C. W. Mak

**Affiliations:** 1 Department of Medicine, The First Affiliated Hospital of Guangzhou Medical University, Guangzhou Institute of Respiratory Health, State Key Laboratory of Respiratory Disease, Guangzhou, China; 2 Department of Medicine, Li Ka Shing Faculty of Medicine, The University of Hong Kong, Hong Kong, SAR China; 3 Department of Pharmacology & Pharmacy, Li Ka Shing Faculty of Medicine, The University of Hong Kong, Hong Kong, SAR China; 4 Research Centre of Heart, Brain, Hormone and Healthy Aging, Li Ka Shing Faculty of Medicine, The University of Hong Kong, Hong Kong, SAR China

**Keywords:** Lipid, Hyperlipidemia, Heart damage, Oxidative stress, Inflammation

## Abstract

**Background:**

Lipid dysregulation is a classical risk factor for cardiovascular disease (CVD), yet scanty evidence existed regarding cardiac lipid metabolism that is directly related to heart damage. Recently, the relationship between dyslipidemia and pro-inflammatory insults has led to further understanding on the CVD-predisposing effects of dyslipidemia. The aims of the present study were to investigate whether high-fat high-cholesterol (HFHC) diet-induced hyperlipidemia would cause heart damage and to study the potential role of local cardiac lipid dysregulation in the occurrence of cellular injury.

**Methods:**

Male Sprague–Dawley rats were divided into normal chow or HFHC diet groups, and sacrificed after 2 or 4 weeks, respectively. Lipid peroxidation marker level was measured. Lipid parameters in the rat hearts were detected. Cardiac damage was evaluated.

**Results:**

HFHC diet increased serum levels of cholesterol and free fatty acids (FFAs) and led to systemic oxidative stress and pro-inflammatory status. Cardiac lipid dysregulation, which was characterized by elevated levels of cholesterol and adipocyte (A)- and heart (H)-fatty acid binding proteins (FABPs), occurred after HFHC diet for 4 weeks. Cardiac damage was further evident with elevated circulating H-FABP levels, increased cardiac interstitial fibrosis and the loss of troponin I.

**Conclusion:**

Our data demonstrated that HFHC diet led to systemic and cardiac lipid dysregulation, accompanied by systemic oxidative and pro-inflammatory stresses, and these may finally cooperate to cause a series of pathological changes of the heart tissue. Our findings suggest that maintenance of lipid regulation may be essential in the prevention of heart damage.

## Background

Dyslipidemia is a well-established risk factor of cardiovascular diseases (CVD) [1]. Accumulating epidemiological and clinical evidence has demonstrated the role of plasma low-density lipoprotein (LDL) and high-density lipoprotein (HDL) in the prediction of cardiovascular events and all-cause deaths in patients with CVD [2]. However, few studies have focused on cardiac levels of these lipid parameters locally.

Cholesterol is an important structural component of cell membranes that plays a major role in maintaining proper membrane permeability and fluidity [3]. Under physiological conditions, cells are prevented from intracellular cholesterol accumulation through precise regulation of synthesis, influx and efflux of cholesterol. 3-Hydroxy-3-methylglutaryl CoA (HMG-CoA) reductase is a key enzyme regulating and limiting the rate of synthesis; cholesterol influx is mediated by LDL receptor and efflux is mainly controlled by ATP-binding cassette transporter A1 (ABCA1) [4, 5]. Factors that compromise these mechanisms may lead to excess accumulation of cholesterol, which may in turn lead to cytotoxicity characterized by enhanced necrosis and apoptosis of cells [6].

Free fatty acids (FFAs) serve as important fuel molecules for myocardial contractions yet elevated FFA levels may also play a central role in pathological processes of CVD [7, 8]. Plasma FFA levels were found to be independently associated with incidence of ischemia heart disease as well as cardiovascular mortality in patients with coronary artery disease (CAD). Moreover, elevated cardiac FFA levels observed in ischemia have been reported to aggravate heart damage and to be proarrhythmic, which may be due to the toxicity of FFAs and their metabolic intermediates [9, 10].

Intracellular lipid chaperons known as fatty acid binding proteins (FABPs) are a group of molecules facilitating FFAs transportation within cells and modulating intracellular lipid metabolism, at least 9 subtypes have already been described, each named after the first tissue of isolation or identification [11, 12]. FABPs were found to be abundant in tissues with high rate of FFA metabolism, such as heart, and parallel lipid oxidation in a number of pathophysiological conditions [13, 14]. Therefore, FABPs may play a major role in preventing intracellular accumulation of excess FFAs and their intermediates by transporting these compounds to their sites for metabolic conversion.

On the other hand, oxidative and inflammatory responses are known to be important pathogenic factors underpinning the occurrence of CVD, and increasing evidence has emphasized the protective role of adiponectin in cardiovascular disorders [15, 16]. Many studies have demonstrated the effects of high-fat diet on inflammatory responses in rodents [17, 18]. However, existing data are still somewhat contradicting, and evidence is sparse for high-fat high-cholesterol (HFHC) diet supplementation.

In the present study, we hypothesized that HFHC diet could influence both lipid parameters and oxidative/inflammatory markers which may together contribute to heart damage. Systemic lipid parameters and oxidative/inflammatory markers, cardiac lipid parameters and morphological changes were examined.

## Methods

### Animal experimental design

Male Sprague–Dawley rats (*n* = 32) weighing about 150 g were fed for 2 days with regular chow diet (RD) (PicoLab Rodent diet 20, Japan SLC Co., Hamamatsu, Japan) before randomly dividing into 2 groups: control and HFHC. The former received normal diet throughout the experiment and the latter was fed with HFHC diet (D12109C, Research Diets, New Brunswick, NJ, USA). Rats were given free access to experimental diets and water, the consumption of diets was measured every day and the gain of body weight was examined every week. The major components of 2 different diets were shown in Table 1. Half of animals were sacrificed at 2 weeks, and the rest at 4 weeks. Arterial blood was obtained by direct heart puncture under sodium pentobarbital anesthesia, rat hearts were rapidly removed and cut into three transversal slices, the middle portion was fixed in 4% paraformaldehyde and embedded in paraffin blocks, top and bottom portions were immediately frozen at -80 °C for future analysis.

**Table 1:** Major components of experimental diets

Ingredient (%)	Regular chow diet (RD)	High-fat high-cholesterol diet (HFHC)
Protein	20.0	22.5
Carbohydrate	52.9	45.0
Fat	10.6	20.0
Cholesterol	0.02	1.3
Sodium cholate	0	0.5
Total energy (kcal/gm)	3.41	4.50

### Preparation of cardiac protein

A small piece of the heart tissue from each rat was homogenized and then total protein was obtained using T-PER Tissue Protein Extraction Reagent (Thermo Scientific, Rockford, IL, USA) according to the protocols provided by the manufacturers. Protein concentration in the supernatant was measured using BioRad protein assay reagent.

### Assessment of lipid peroxidation marker level

Serum malondialdehyde (MDA) levels were assayed using TBARS assay kit (Cayman, Ann Arbor, MI, USA) according to the manufacturer’s instructions.

### Measurement of CINC-1 and adiponectin levels

Serum levels of cytokine-induced neutrophil chemoattractant-1 (CINC-1) (R&D Inc. Minneapolis, MN) and total adiponectin (Invitrogen, Carlsbad, USA) were analyzed using. ELISA kits according to the manufacturer’s instructions.

### Measurement of lipid parameters

Serum cholesterol, triglyceride and FFA levels were measured with kits from BioAssay Systems (Hayward, CA, USA). Lipid extraction of the heart was performed according to the established protocol as modified by the company. Briefly, heart tissues were homogenized in lipid extraction buffer prepared with 5 volume isopropanol, 2 volume water and 2 volumes of Triton X-100. After vigorously vortexing, the homogenates were centrifuged 5 min at 14,000 rpm. Supernatants were collected to examine lipid levels mentioned above (BioAssay Systems).

### Western blotting analysis

Aliquots were separated by SDS-polyacrylamide gel, and then transferred onto a polyvinylidene fluoride (PVDF) membrane (Bio-Rad Laboratories, Hercules, CA, USA). After blocking with 5% (*w*/*v*) non-fat milk in Tris Buffered Saline containing 0.5% (*v*/v) tween-20 (TBST), membranes were then incubated with antibodies to HO-1 (Stressgen, Victoria, BC, Canada), cleaved caspase-3, caspase-3, Bax, Bcl2 (Cell Signaling, Danvers, MA, USA), A-FABP, H-FABP (Santa Cruz), HMG-CoA reductase (Millipore), E-FABP (Biovision, California, USA), ATP-binding cassette transporter-1 (ABCA1), LDL receptor (Novus Biologicals, Littleton, CO, USA) overnight at 4 °C. The membranes were later incubated with horseradish peroxide-conjugated goat anti-rabbit, goat anti-mouse, or mouse anti-goat antibody (Dako, Danmark) for 2 h at room temperature. Afterwards, membranes were probed with enhanced chemiluninescence (ECL plus) (Amersham, Piscataway, UK). GAPDH (Santa Cruz Bioechnology) was used as loading control and densitometric analysis of the bands was performed with GeneTools (Syngene, Frederick, MD, USA).

### Serum markers of cardiac damage

Serum levels of H-FABP (Hycult biotech Inc., PA, USA) and cardiac troponin I (Life Diagnostics Inc., PA, USA) were measured as markers of cardiac damage according to the manufacturer’s instructions.

### Histological staining

Sirius Red staining was conducted to examine interstitial fibrosis within heart tissue, and troponin I (Santa Cruz) staining was performed as a gold standard for myocardial damage. For each section, positive staining area were calculated on 9 randomly chosen digitalized photographs taken by AxioCam HRc microscope camera (Carl Zeiss) connected with Zeiss Axioskop 2 plus microscope. Two sections were observed for each animal and each group contained 5 animals. Results were calculated as a proportion of staining area to the total area and expressed as fold change (Sirius Red) or percentage (Troponin I) versus control.

### Statistical analysis

Data were expressed as means ± SEM. Comparisons between two groups (IH and control at 2 and 4 weeks, respectively) were performed with Student’s t test using GraphPad Prism 5.0 (GraphPad Software Inc., San Diego, CA, USA). A *p* < 0.05 was considered statistically significant.

## Results

### Metabolic data of experimental groups

There was a trend of body weight increase in HFHC groups despite less food consumption each day. HFHC diet resulted in significant increases in serum total cholesterol reflected by suppressed HDL-C levels and elevated LDL-C levels and in serum FFA levels. In the heart tissue, HFHC diet elevated cholesterol levels at 4 weeks without any effect on FFA levels at any time point (Table 2).

**Table 2:** Metabolic data of experimental groups

	Control 2w	HFHC 2w	Control 4w	HFHC 4w
Body weight(g)	279.7 ± 5.7	279.1 ± 6.2	399.4 ± 11.5	415.0 ± 19.3
Food consumption (g/day)	25.64 ± 0.4	21.97 ± 0.3^***^	25.64 ± 0.4	21.97 ± 0.3^***^
Serum cholesterol (mg/dl)	26.6 ± 4.3	100.0 ± 3.8^***^	34.14 ± 2.4	112.1 ± 4.6^***^
Serum HDL (mg/dl)	19.1 ± 2.2	6.2 ± 0.4^***^	20.0 ± 1.7	9.3 ± 1.4^***^
Serum LDL (mg/dl)	14.6 ± 2.0	93.8 ± 3.9^***^	15.2 ± 2.1	102.9 ± 3.6^***^
Serum FFA (μM)	63.8 ± 4.0	107.5 ± 9.1^**^	98.0 ± 5.8	114.6 ± 4.9^*^
Serum MDA (μM)	40.7 ± 2.7	48.9 ± 3.5	42.0 ± 2.8	59.3 ± 5.0^*^
Serum CINC-1 (pg/ml)	74.4 ± 11.0	139.1 ± 20.7^*^	103.4 ± 12.5	250.6 ± 28.8^***^
Serum adiponectin (μg/ml)	10.3 ± 1.2	7.5 ± 0.6^*^	8.1 ± 0.6	4.0 ± 0.4^***^
Cardiac cholesterol (mg/g tissue)	0.16 ± 0.01	0.19 ± 0.02	0.15 ± 0.009	0.19 ± 0.01^*^
Cardiac FFA (mg/g tissue)	25.4 ± 2.6	21.4 ± 1.8	35.7 ± 4.9	28.0 ± 3.5

*HFHC* high-fat-high cholesterol, *HDL* high-density lipoprotein, *LDL* low-density lipoprotein, *FFA* free fatty acids, *MDA* malondialdehyde, *CINC-1* cytokine-induced eutrophil chemo attractant 1. Values were expressed as means±SEM. *N* = 8 for each group; *p < 0.05, **p < 0.01, ***p < 0.001 vs controls

### HFHC diet mediates inflammation

**Fig. 1:**
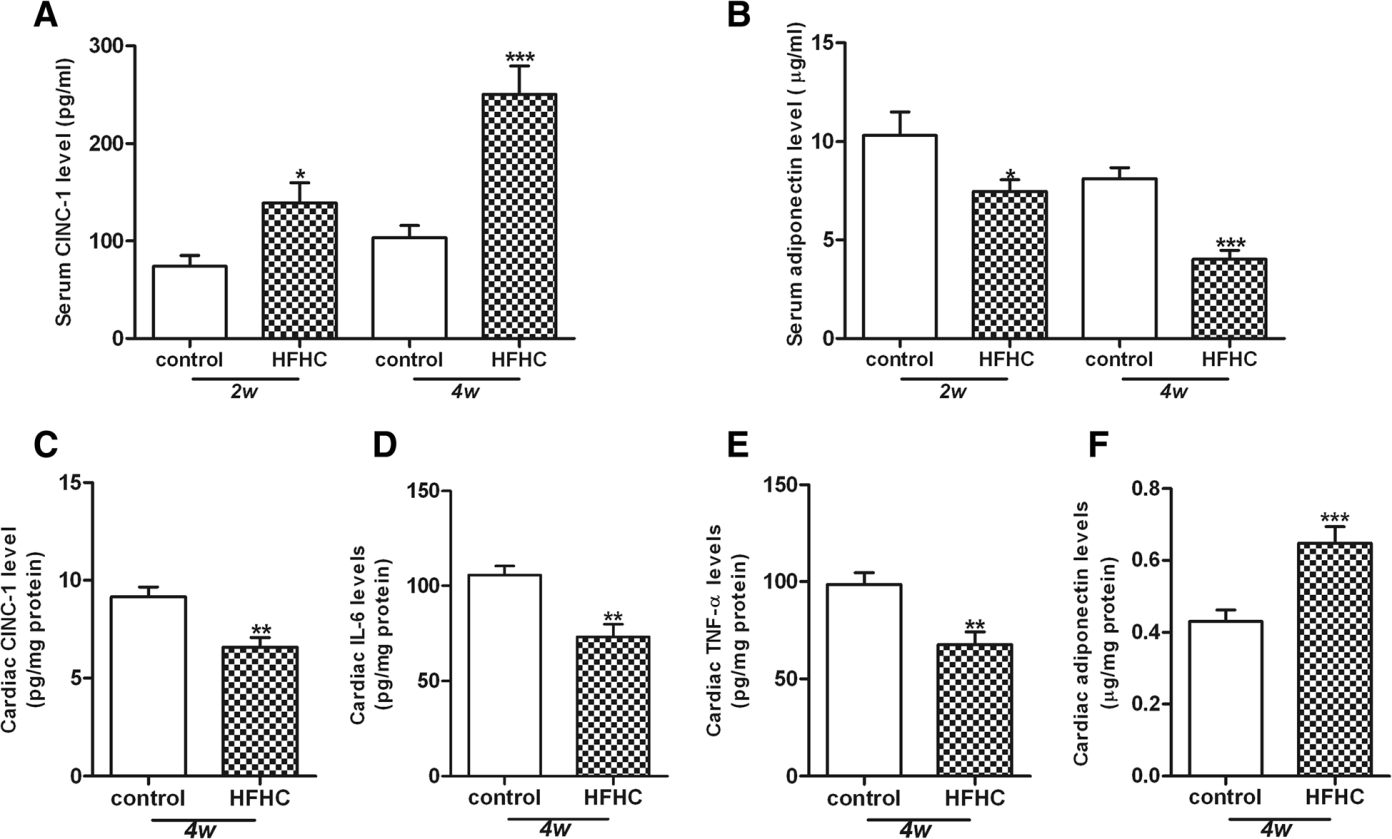
Serum and cardiac inflammatory markers. HFHC diet time-dependently elevated CINC-1 level (**a**) and reduced adiponectin level in the serum (**b**). HFHC diet at 4 weeks reduced the cardiac level of CINC-1 (**c**), IL-6 (**d**) and TNF-α (**e**) and increased cardiac adiponectin level (**f**). Values were expressed as means±SEM and results were calibrated with protein concentration for cardiac markers (*n* = 8). *p < 0.05, **p < 0.01, ***p < 0.001 vs. relative controls

It has reported that CINC-1, IL-6 and TNF-α are the major pro-inflammatory cytokines whereas adiponectin is an anti-inflammatory chemokine [19, 20]. To examine whether HFHC diet would mediate inflammation, we thus measured the systemic and cardiac levels of CINC-1, IL-6, TNF-α and adiponectin in the rats of HFHC diet. HFHC diet significantly elevated CINC-1 levels but reduced serum adiponectin levels at 2 and 4 weeks, respectively (Fig 1a, b). The levels of CINC-1, IL-6, TNF-α in the heart tissue were greatly decreased in the HFHC group compared with the control group at 4 weeks (Fig. 1c, d, e). In contrast, the level of adiponectin in the heart tissue was much higher in the HFHC group than the control group (Fig. 1f). However, serum levels of IL-6 and TNF-α were below the detectable range of the commercially available kits.

### HFHC diet regulates cholesterol metabolism in the heart

**Fig. 2:**
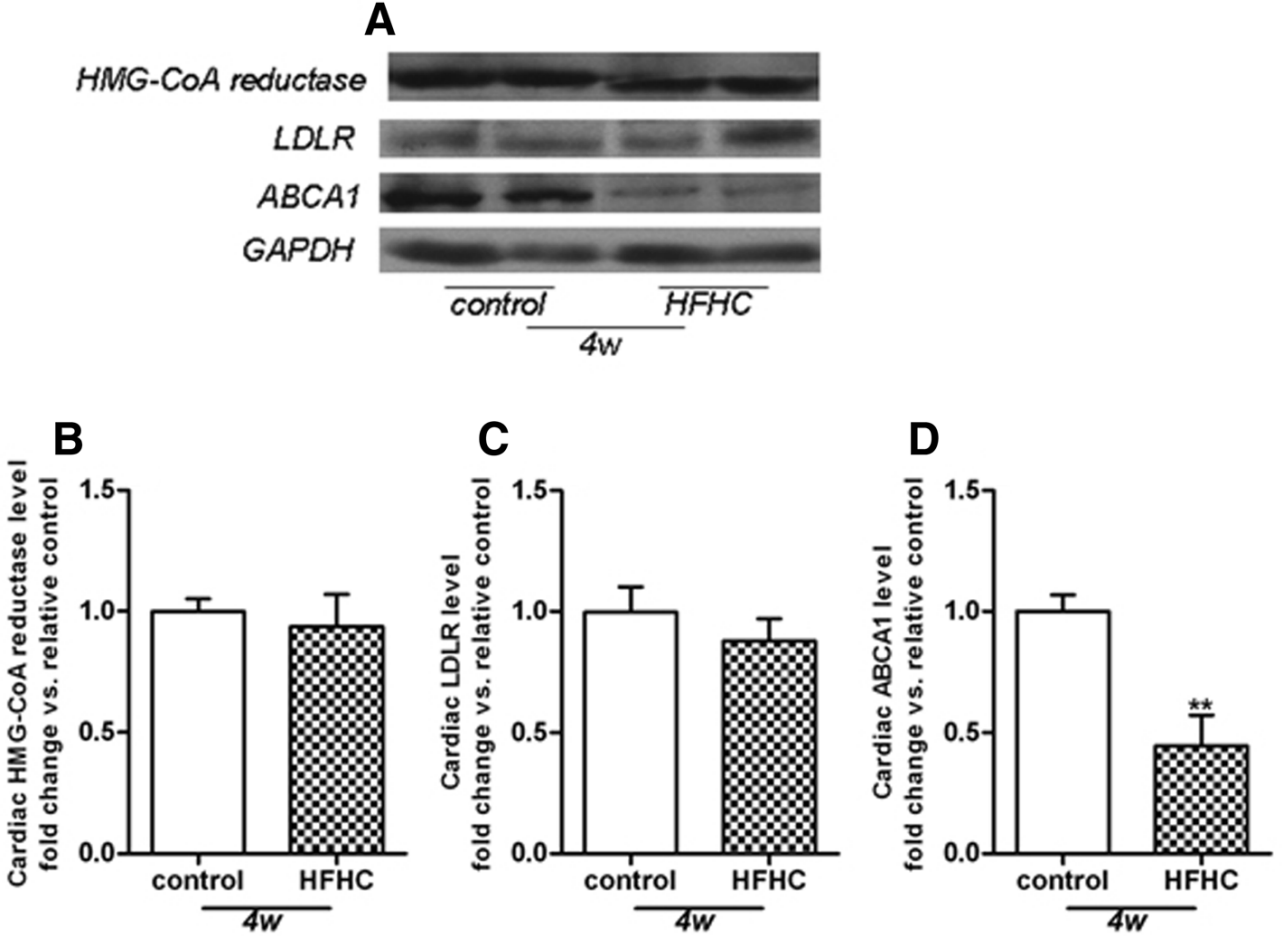
Cardiac expression of proteins regulating cholesterol metabolism. Representative Western Blot pictures were shown in (**a**). HFHC diet had no effect on protein expression levels of HMG-CoA reductase (**b**) and LDLR (**c**), with significant suppression of ABCA1 protein levels (**d**) in heart homogenates. Protein levels were normalized to GAPDH levels and results were expressed as fold change vs. control. **p < 0.01 vs. relative control at 4 weeks

Cardiac expression of proteins regulating cholesterol metabolism was detected by western blotting. The results demonstrated that HFHC diet had no effect on expression levels of HMG-CoA reductase (Fig. 2a, b) and LDLR (Fig. 2a, c) in the heart tissue. In contrast, the protein level of ABCA1 was dramatically reduced in the HFHC group compared with the control group (Fig. 2a, d).

### HFHC diet activates the apoptosis in the heart

**Fig. 3:**
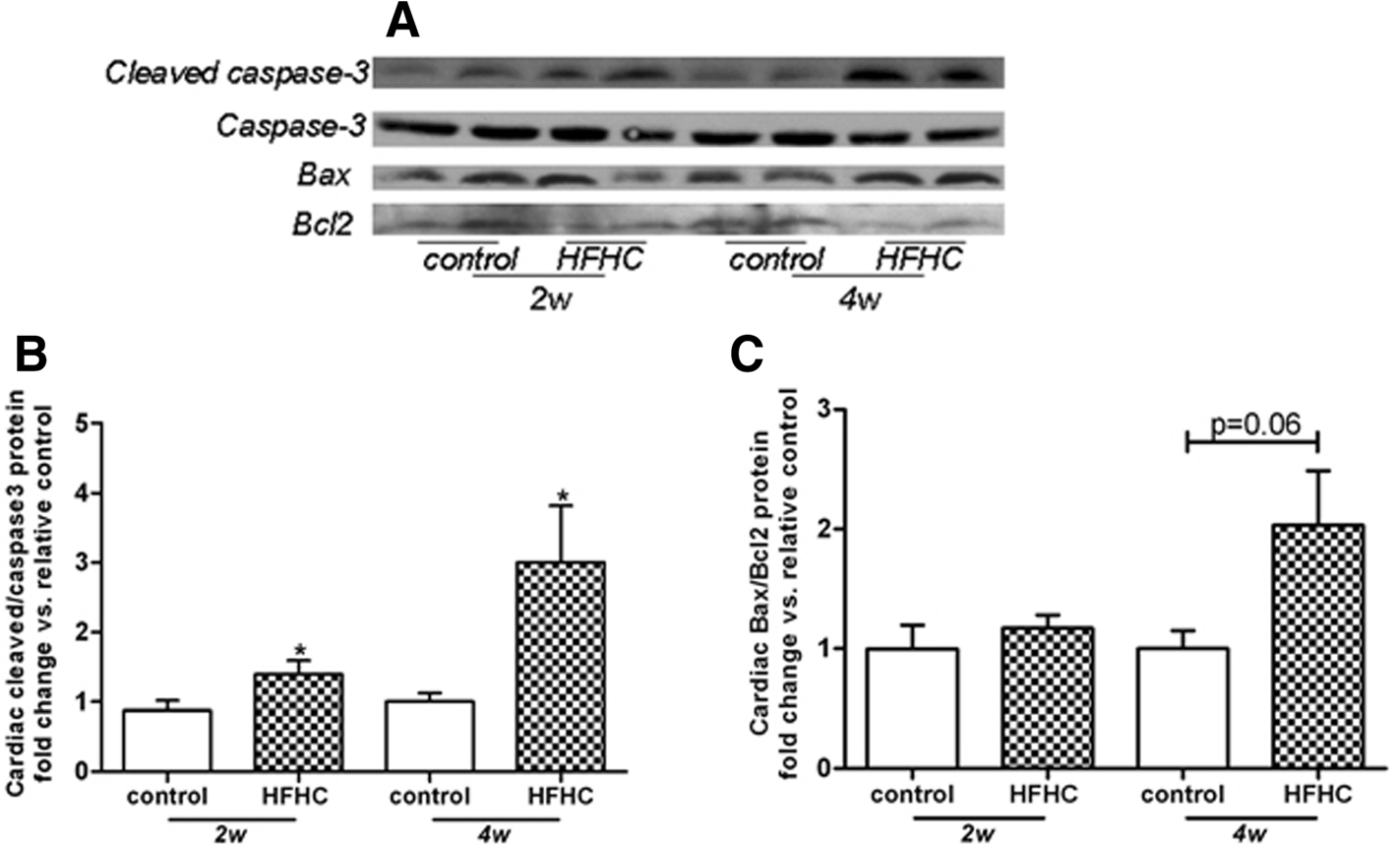
HFHC diet activates the apoptosis in the heart. Representative Western Blot pictures were shown in (**a**). HFHC diet time-dependently raised cleaved caspase-3/caspase-3 ratio (**b**), with a non-significant elevation of Bax/Bcl2 ratio at 4 weeks (**c**) in heart homogenates. Values were expressed as fold change vs. control. *p < 0.05 vs. relative controls

Next, we examined the apoptosis in the heart tissue. The results showed that HFHC diet raised cardiac cleaved /intact caspase-3 ratio in a time dependent manner (Fig. 3a, b), indicating the activation of caspase-3 and initiation of apoptosis. Furthermore, HFHC diet also resulted in a trend of elevation of cardiac Bax/Bcl2 ratio at 4 weeks (Fig. 3a, c).

### Cardiac expressions of FABPs

**Fig. 4:**
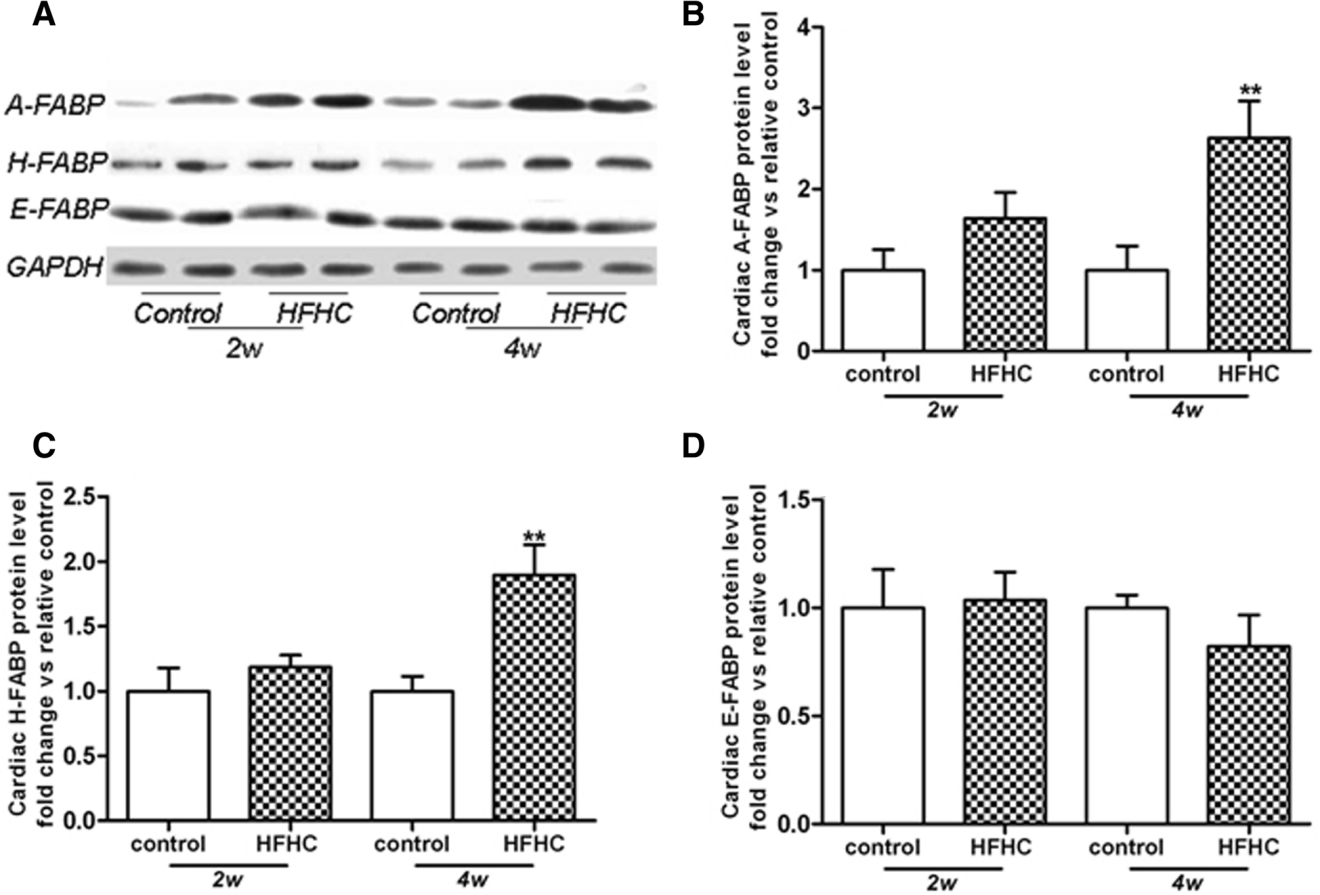
HFHC diet regulates the cardiac protein levels of FABPs. Representative Western Blot pictures were shown in (**a**). HFHC diet significantly enhanced protein expression levels of A- (**b**) and H-FABPs (**c**) at 4 weeks but no change detected for E-FABP levels in heart homogenates (**d**). Protein levels were normalized to GAPDH levels and results were expressed as fold change vs. control. **p < 0.01 vs. relative controls

Next, we sought to determine the effects of HFHC diet on FABPs in the heart tissue. Three subtypes (A-, H- and E-FABPs) were detected by western blotting. The protein expressions of A- (Fig. 4a, b) and H-FABP (Fig. 4a, c) were markedly enhanced in the HFHC group compared with the control group at 4 weeks. However, there was no difference of E-FABP between the control group and the HFHC group at 4 weeks (Fig. 4a, d).

### HFHC diet induces heart injury

**Fig. 5:**
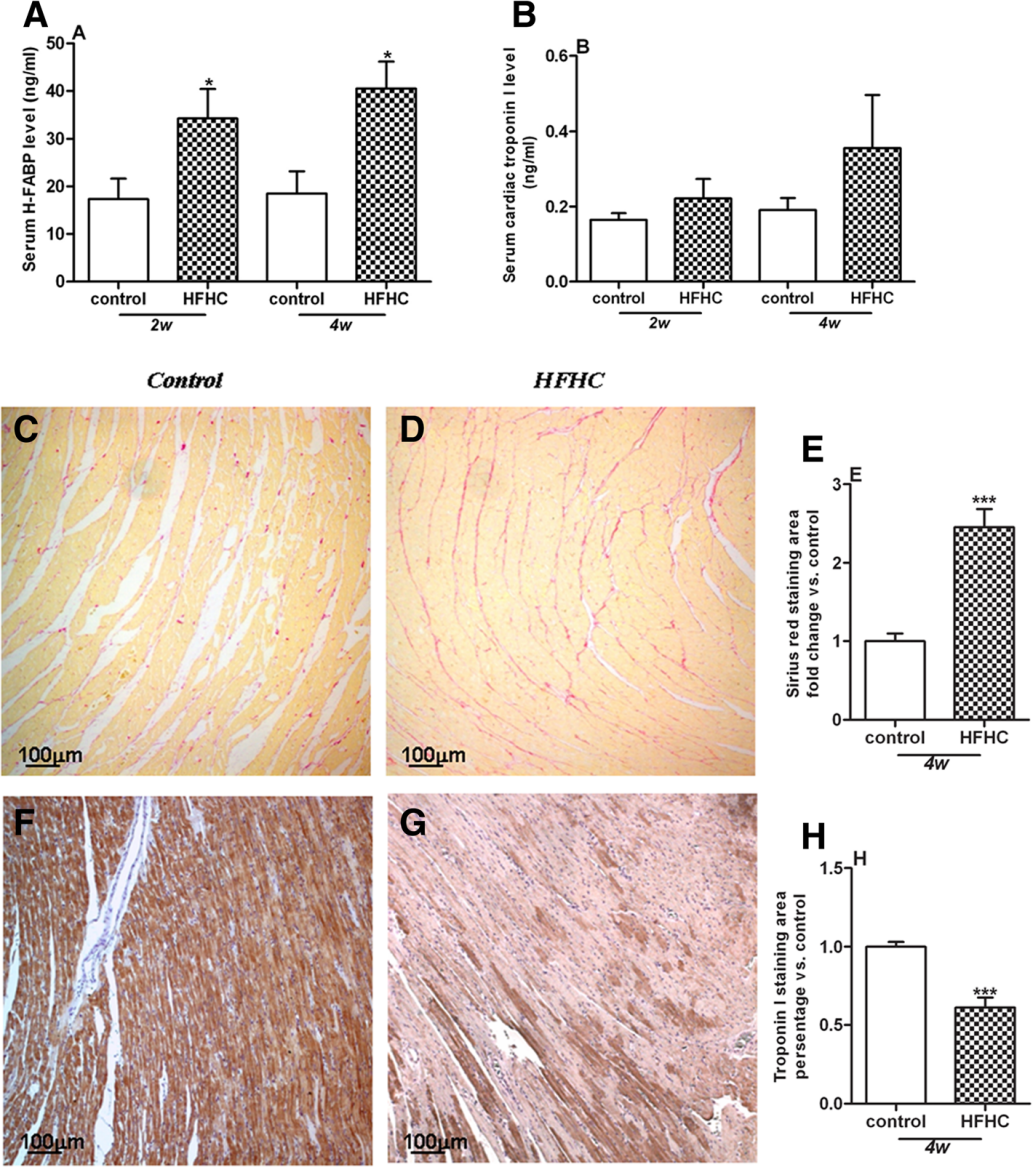
HFHC diet induces heart damage. HFHC diet time-dependently increased serum H-FABP levels (**a**) but not serum troponin I levels (**b**). Histological evaluation heart tissue was conducted at 4 weeks. Sirius red and troponin I staining showed increased interstitial fibrosis (**e**) and loss of troponin I (**h**) in heart sections of HFHC group, respectively. Staining area of Sirius red and troponin I was expressed as fold change and percentage vs. control, respectively. Representative pictures were shown as (**c**-**d**) for Sirius red, and (**f**-**g**) for troponin I stainings. *p < 0.05, ***p < 0.001 vs control.

Heart damage was evaluated by serological markers as well as histological change in the heart. HFHC diet led to a time-dependent increase of serum H-FABP levels (Fig. 5a). There was no difference of serum cardiac troponin I levels between the control group and HFHC group (Fig. 5b). Histological evaluation of heart tissue was conducted at 4 weeks. Sirius red staining showed that compared with the control group, interstitial fibrosis was greatly increased in the HFHC group (Fig. 5c, d, e). Moreover, troponin I staining demonstrated depletion of myocardial striation in the heart tissues of HFHC group (Fig. 5f, g, h).

## Discussion

There are three major findings derived from the present study. First, HFHC diet raised cardiac levels of total cholesterol and expressions of A-/H-FABPs along with elevated serum levels of total cholesterol and FFA. Second, HFHC diet led to systemic oxidative and pro-inflammatory stresses. Third, HFHC diet resulted in heart damage reflected by elevated serum levels of H-FABP and a series of morphological changes.

Dyslipidemia is a well-known risk factor of cardiovascular disorders [1]. Both LDL and HDL cholesterol levels were demonstrated to be strongly associated with CVD independent of other risk factors [21]. Here, we found that HFHC diet for 2 and 4 weeks led to a significant elevation of serum total cholesterol level, further reflected by enhanced LDL and suppressed HDL levels. These data suggested that HFHC diet may cause systemic hypercholesterolemia that was prone to increased cardiovascular risk. Moreover, the HFHC diet-induced elevation of cardiac cholesterol levels at 4 weeks indicates that HFHC diet may directly lead to myocardial damage via excess cholesterol accumulation within heart tissue.

Normal cells are protected from excess cholesterol accumulation via multiple steps including cholesterol synthesis, influx and efflux [22, 23], which may be compromised by various pathophysiological conditions. Enhanced mRNA expression levels of HMG- CoA reductase and LDL-R have been described in kidney of FVB^db/db^ mice developing severe diabetic nephropathy, leading to renal cholesterol accumulation [24]. Free cholesterol loading of macrophages was found to induce increased degradation of ABCA1, thus resulting in further cholesterol accumulation [25, 26]. In this study, HFHC diet suppressed ABCA1 levels in the heart without any effect on HMG-CoA reductase and LDL-R, suggesting that impaired cholesterol efflux might be responsible for HFHC diet-induced cardiac cholesterol accumulation.

Numerous studies regarding cytotoxity of intracellular cholesterol accumulation have concentrated on macrophages [27, 28]. Widespread mitochondrial dysfunction and cell apoptosis have been found in free cholesterol-loaded macrophages [29], with increased levels of proapoptotic protein Bax. We herein demonstrated a time-dependent HFHC diet-induced elevation of the cleaved form of caspase-3, accompanied by a borderline increase of Bax/Bcl2 ratio at 4 weeks. These data suggested that HFHC diet led to the acceleration of cell apoptosis within the heart tissue, which may be partly mediated through the intrinsic apoptosis pathway. Moreover, the parallel increase of cell apoptosis may strongly suggest a detrimental role of cardiac cholesterol accumulation in cell injury.

FFAs serve the body as physiologically important energy substrates through oxidation, yet high concentrations of FFAs have been demonstrated to be a risk factor for cardiovascular disease [8, 30]. Plasma FFA levels were reported to be increased in patients with angiographic CAD and be associated with severity of heart failure [9]. Cardiomyocytes generate up to 70% of their energy requirements through β-oxidation of FFAs [31, 32]. However, the elevated cardiac levels of FFAs were prone to be harmful to the heart in multiple ways [33]. Due to the amphiphilic and detergent-like properties, FFAs could disturb ion channels and membrane integrity [34], uncouple mitochondrial enzymes, thereby reducing the efficiency of respiratory cycle and compromising contractile function of the heart. In this study, HFHC diet resulted in a time-dependent increase of serum FFAs without any effect on cardiac levels of FFAs, suggesting a protective mechanism that may act in preventing excess accumulation of FFAs within the heart tissue.

FABPs may greatly contribute to sequester FFAs accumulation and prevent tissue damage, given their roles in facilitating mobilization of FFAs to different cellular compartments. Among 3 subtypes of FABPs (A-, H-and E-FABP) found in the heart tissue, HFHC diet caused a non-significant increase in A- and H-FABP protein levels at 2 week but reaching significant levels at 4 weeks. While it may initially be protective in preventing cardiac accumulation of FFAs with elevated circulating FFAs levels, the upregulation of A- and H-FABPs may be potentially detrimental. Firstly, the elevation of FABPs would enhance β-oxidation of cardiac FFAs in parallel, which was shown to impair heart function because of “oxygen wastage” [32]. Moreover, the compensatory elevation of FABPs might be dampened during longer period of HFHC diet supplementation [35], thereby leading to excess lipid accumulation with further heart damage.

In consistent with previous studies [36, 37], we found that HFHC diet caused a time-dependent elevation of systemic MDA and CINC-1 levels. Moreover, HFHC diet suppressed serum levels of adiponectin independent of weight gain, indicating the direct effect of dyslipidemia on hypo-adiponectinemia. In contrast, the pro-inflammatory markers including the levels of CINC-1, IL-6 and TNF-α were reduced while adiponectin level was elevated in the heart tissues of HFHC diet rats. These observations might be due to local compensatory protective effects at the early stage of HFHC diet. After long term HFHC diet, these cardioprotective responses might be reduced and reversed. Since the depletion of adiponectin in mice was demonstrated to increase cardiomyocyte apoptosis and interstitial fibrosis [38], it could be speculated that hypo-adiponectinemia induced by HFHC diet treatment may also account for the pathological changes observed in heart tissue. Taken together, the relationship between dyslipidemia and oxidative/inflammatory responses may lead to further understanding on the mechanisms of lipid dysregulation in the pathogenesis of cardiovascular events.

Finally, heart damage was evaluated by serological markers and histological changes. HFHC diet caused a time-dependent increase in serum H-FABP levels with a non-significant elevation in serum cardiac troponin I levels, reflecting higher sensitivity and specificity of H-FABP in the prediction of cardiomyocyte injury compared with other biochemical markers [39]. In support serum H-FABP but not troponin I levels have been found to be elevated in obstructive sleep apnea (OSA) patients without symptomatic CVD compared to those without OSA, suggesting that H-FABP may serve as a marker in the early detection of cardiac injury in OSA patients.

Histological staining revealed that HFHC diet at 4 weeks led to increased cardiac interstitial fibrosis with depleted myocardial striation, suggesting the loss of contractile activity of myocardium, which would eventually lead to cardiac dysfunction and heart failure. Future studies will be required on the measurements of hemodynamic parameters for cardiac function in this model. Despite our illustration of systemic and cardiac lipid dysregulaiton after HFHC diet supplementation, our current findings should be interpreted within the constraints of potential limitations. First, species differences preclude any direct translation to human subjects, although results in this animal model are somehow consistent with human observations. Second, further studies with drug intervention may be required to investigate the causal relationship between cardiac lipid dysregulation and pathological changes in the heart. Third, although we found that cholesterol accumulation was increased in the heart accompanied by enhanced cardiomyocyte apoptosis and fibrosis, the potential mechanisms underlying cholesterol accumulation that results in the loss of troponin I and fibrosis have not been explored in the current study. Other factors such as hyperlipidemia, insulin resistance or oxidative stress may also be involved, which need further investigation.

## Conclusion

This study demonstrated that HFHC diet for 4 weeks caused cardiac lipid dysregulation characterized by cholesterol accumulation and increased A-/H-FABPs within heart tissue, as well as enhanced systemic oxidative and inflammatory stresses. We propose that these may cooperate to result in heart damage observed at 4 weeks. Although our present study has shed some light on the mechanisms to link diet-induced dyslipidemia and myocardial injury, the pathogenesis of cardiac morphological changes and contractile dysfunction still deserves further in-depth investigation.

## Abbreviations

ABCA1: ATP-binding cassette transporter A1
CINC-1: Cytokine-induced neutrophil chemo attractant-1
CVD: Cardiovascular disease
FABPs: Fatty acid binding proteins
FFAs: Free fatty acids
HDL: High-density lipoprotein
HFHC: High-fat high-cholesterol
HMG-CoA: 3-Hydroxy-3-methylglutaryl CoA
LDL: Low-density lipoprotein
MDA: Malondialdehyde
